# Strongly enhanced luminescence of Sr_4_Al_14_O_25_:Mn^4+^ phosphor by co-doping B^3+^ and Na^+^ ions with red emission for plant growth LEDs

**DOI:** 10.1039/c7ra11967d

**Published:** 2018-01-04

**Authors:** Jiaqi Long, Xuanyi Yuan, Chaoyang Ma, Miaomiao Du, Xiaoli Ma, Zicheng Wen, Ran Ma, Yuzhen Wang, Yongge Cao

**Affiliations:** Beijing Key Laboratory of Opto-electronic Functional Materials & Micro-nano Devices, Department of Physics, Renmin University of China Beijing 100872 China caoyongge@ruc.edu.cn

## Abstract

Development of a more cost-effective radiation source for use in plant-growing facilities would be of significant benefit for commercial crop production applications. A series of co-doped B^3+^ and Na^+^ ions Sr_4_Al_14_O_25_:Mn^4+^ inorganic luminescence materials which can be used for plant growth were successfully synthesized through a conventional high-temperature solid-state reaction. Powder X-ray diffraction was used to confirm the crystal structure and phase purity of the obtained samples. Then scanning electron microscopy elemental mapping was undertaken to characterize the distribution of the doped ions. Detail investigations on the photoluminescence emission and excitation spectra revealed that emission intensity of tetravalent manganese ions can be well enhanced by monovalent sodium ions and trivalent boron ions under near-ultraviolet and blue excitation. Additionally, crystal field parameters and energies of states are calculated and discussed in detail. Particularly we achieve a photoluminescence internal quantum yield as high as 60.8% under 450 nm blue light excitation for Sr_4_Al_14_O_25_:Mn^4+^, Na^+^, B^3+^. Therefore, satisfactory luminescence properties make these phosphors available to LEDs for plant growth.

## Abstract 2

(a) Emission spectrum of LEDs fabricated with 445 nm blue chip and Sr_2_Si_5_N_8_:Eu^2+^ red phosphor. (b) Emission spectrum of LEDs fabricated with 445 nm blue chip and Sr_4_Al_14_O_25_:Mn^4+^ phosphor. (c and d) Absorption spectrum of chlorophyll-b and chlorophyll-a.

## Graphical abstract

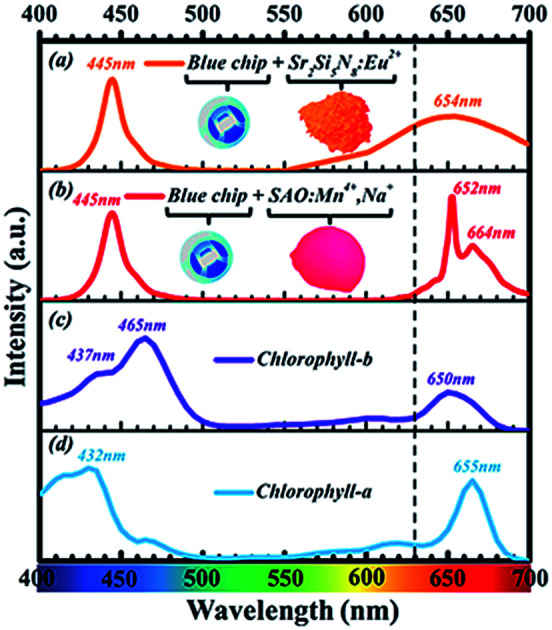

## Introduction

It is known that green plants can be grown only using red and blue monochromatic light, because chlorophyll has its second distinct absorption peak in the vicinity of 450 nm (the blue light region) and its first peak in the vicinity of 660 nm (red light region). The blue light is indispensable to the morphologically healthy plant growth, and the red light contributes to the plant leaf photosynthesis.^1,2^ The first successful plant growth experiment using only blue and red LEDs were achieved in June 1994 by Okamoto and Yanagi.^3^

With LEDs price reduced, LEDs are gradually used as plant lighting source, because of its high light efficiency, energy saving and other characteristics. As the driving currents of the blue chip and the red chip are inconsistent, the design of the driver circuit of LEDs for planet growth would be very complex, resulting in increased cost. Therefore, the plant-grown red lamp that is currently in widespread use in the marketplace is composed of a red phosphor rather than a red chip. The most commonly used red phosphors are nitride phosphor (*e.g.*, Sr_2_Si_5_N_8_:Eu^2+^, CaAlSiN_3_:Eu^2+^) because of high luminous efficiency. But their high temperature (∼1600 °C), high pressure (1–10 MPa) preparation conditions and expensive raw materials containing rare earth elements lead to high cost.^4^

**Fig. 1:**
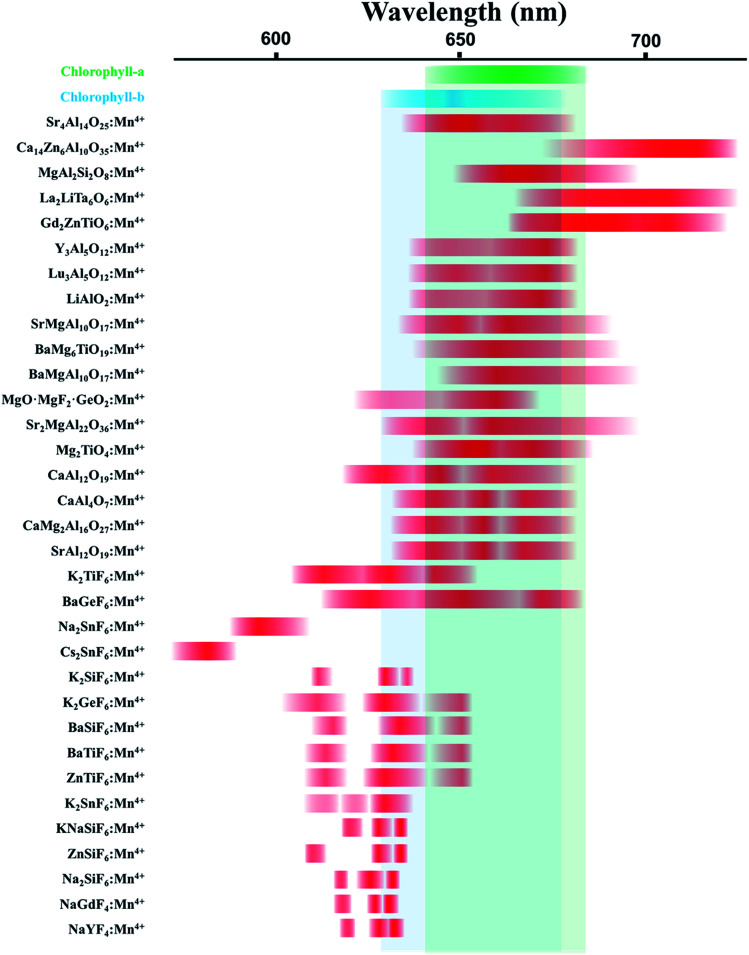
Spectroscopic range of Mn^4+^ ions in various crystals. Green and blue color indicate the absorption spectrum region of chlorophyll-a and chlorophyll-b, respectively. Red color indicates the emission spectrum region of various phosphors with Mn^4+^ ions doped.

In recent years, Mn^4+^-activated fluoride compounds, as an alternative to commercial (oxy)nitride phosphors, are emerging as a new class of non-rare-earth red phosphors for high-efficacy warm white LEDs^5^ compared to Mn^4+^-activated fluoride phosphor, the emission wavelength of Mn^4+^-activated oxide phosphors, such as Sr_4_Al_14_O_25_:Mn^4+^, Y_3_Al_5_O_12_:Mn^4+^, *etc.*, is much longer which is very suitable for plant lighting, because the absorption peak of plant chlorophyll is near 660 nm. The following diagram shows spectroscopic range of Mn^4+^ ions in various crystals.^6^ We can see from Fig. 1 that the emission peak of the Mn^4+^-activated fluoride phosphor and the absorption peak of chlorophyll rarely overlap, while the emission peak of the Mn^4+^-activated oxide phosphors, such as Sr_4_Al_14_O_25_:Mn^4+^, overlap with the absorption peaks of chlorophyll-a and chlorophyll-b.

**Fig. 2:**
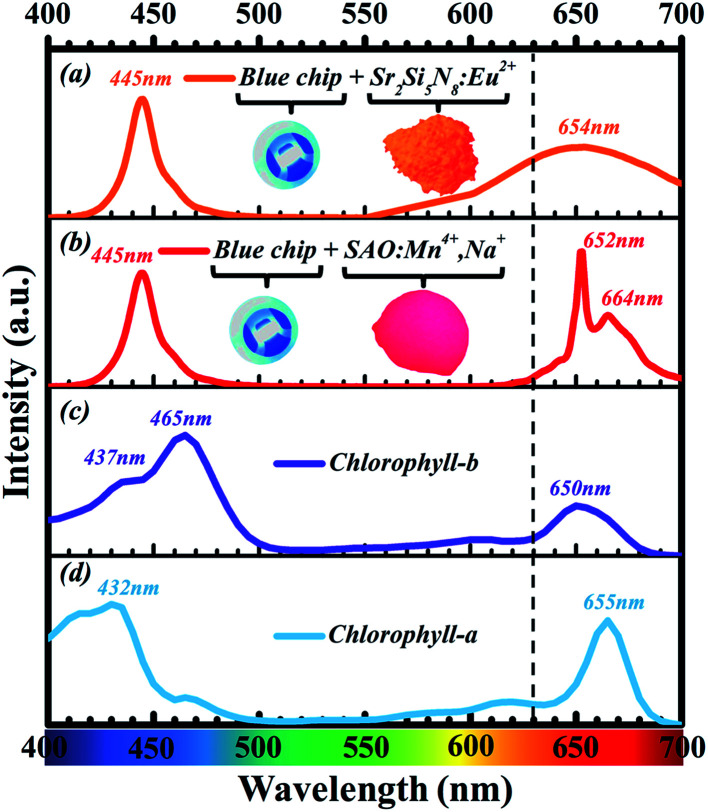
(a) Emission spectrum of LEDs fabricated with 445 nm blue chip and Sr_2_Si_5_N_8_:Eu^2+^ red phosphor. (b) Emission spectrum of LEDs fabricated with 445 nm blue chip and Sr_4_Al_14_O_25_:Mn^4+^, red phosphor. (c) Absorption spectrum of chlorophyll-b. (d) Absorption spectrum of chlorophyll-a (redrawn from Zscheile and Comar's (1941) original data^7^).

As shown in Fig. 2, the emission spectrum of Sr_4_Al_14_O_25_:Mn^4+^ red phosphor has overlap more efficiently with the absorption spectrum of chlorophyll compared with Sr_2_Si_5_N_8_:Eu^2+^ red commercial phosphors. However, most Mn^4+^-activated oxide phosphors cannot be excited effectively by blue light, which limited its application on blue chip-based LEDs. There are some methods to improve the luminous efficiency and luminous intensity of Mn^4+^-activated oxide phosphors, such as impurity doping with Mg^2+^ ions,^8,9^ impurity doping with Bi^3+^ ions, and impurity doping with Na^+^ ions.^10–12^ The objective of this work is to develop a red emitting phosphor which can match with blue chip for possible application in plant growth. In this article, the red phosphors Sr_4_Al_14_O_25_:Mn^4+^, Na^+^, B^3+^ with strong absorption at blue region were synthesized. The difference with Sr_4_Al_14_O_25_:Mn^4+^, Na^+^ phosphors reported by Lili Meng^12^ was that the excitation intensity of the phosphor we prepared was significantly improved, especially in the blue light region. We also found that the doping amount of B^3+^ ions was very crucial. Specific doping ratio of B^3+^ ions and Na^+^ ions make the luminous performance of the phosphor significantly improved. The advancements of current work include significant improvement of luminescent efficiency of Sr_4_Al_14_O_25_:Mn^4+^ by doping B^3+^ and Na^+^ ions. Notably, doping of B^3+^ and Na^+^ ions can improve its visible light excitation efficiency in the spectral range of 400–500 nm so that it can be incorporated as a red component into blue chip-based LED applications for plant growth.

## Experimental section

### Materials and synthesis

Polycrystalline phosphors with composition of Sr_4_Al_14_O_25_:Mn^4+^, Na^+^, B^3+^ were prepared with a high-temperature solid-state reaction. Briefly, the constituent raw materials SrCO_3_ (A. R., 99.9%), Al_2_O_3_ (A. R., 99.99%), Na_2_CO_3_ (A. R., 99%), H_3_BO_3_ (A. R., 99%) and MnO_2_ (A. R., 99.99%) were weighed according to the stoichiometric ratio. Individual batches of 10 g were weighted according to the designed stoichiometry and mixed homogeneously with the same mass of absolute ethyl alcohol as the dispersant. After planetary ball-milling process, the obtained homogeneous slurry was placed in a Petri dish and dried in an oven. Then, the dried mixtures were put into a crucible with a lid and heated in a tubular furnace at 1400 °C for 6 hours in the air. When cooled down to room temperature, the prepared phosphors were crushed and ground for subsequent measurements.

### Characterization

All crystal structure compositions were checked for phase formation by using powder X-ray diffraction (XRD) analysis with a Rigaku X-ray diffractometer (Tokyo, Japan) with a graphite monochromator using Cu Kα radiation (*λ* = 1.54056 Å), over the angular range 10° < 2*θ* < 80°, operating at 40 kV and 40 mA. The schematic crystal structure of Sr_4_Al_14_O_25_ was drawn in VESTA.^13^ The photoluminescence (PL) and photoluminescence excitation (PLE) spectra of the samples were analyzed by using a Hitachi F-7000 spectrophotometer (Tokyo, Japan) with a 150 W Xe lamp.

## Results and discussion

### Microstructure

**Fig. 3:**
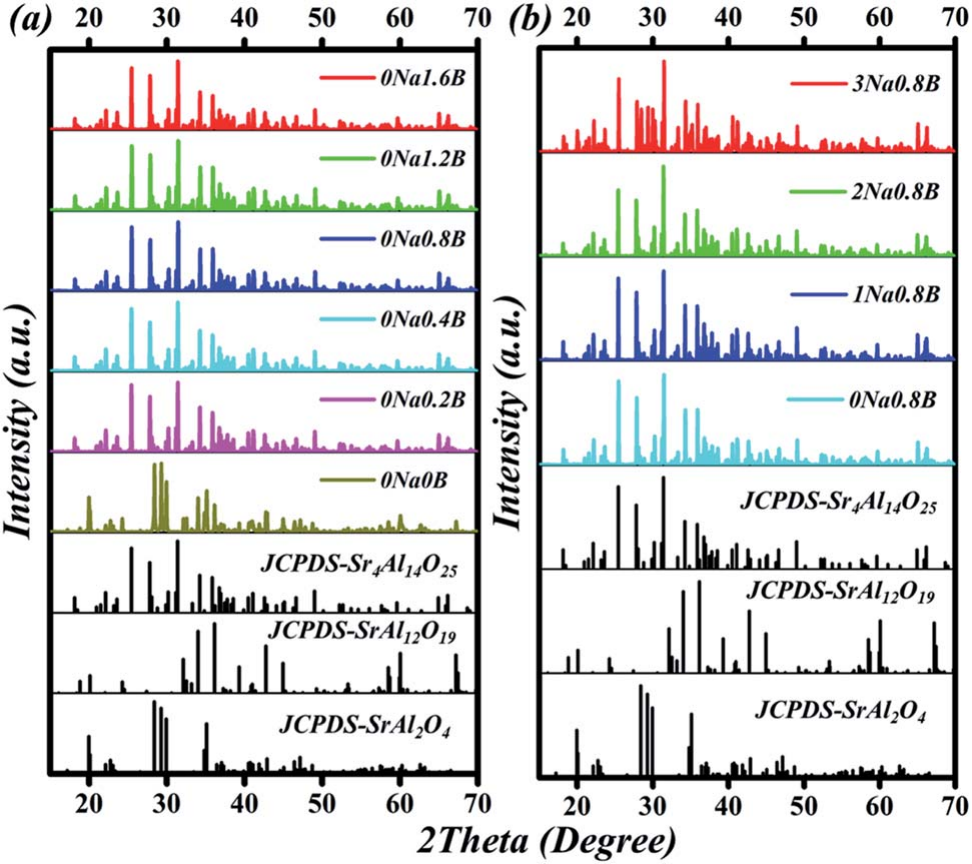
(a) XRD patterns of Sr_4_Al_14_O_25_:0.014Mn^4+^, *x*B^3+^, 0Na^+^ (0 ≤ *x* ≤ 1.6) phosphors. (b) XRD patterns of Sr_4_Al_14_O_25_:0.014Mn^4+^, 0.8B^3+^, *y*Na^+^ (0 ≤ *y* ≤ 3). The reference spectra of Sr_4_Al_14_O_25_ (JCPDS-No. 52-1876), SrAl_12_O_19_ (JCPDS-No. 80-1195), SrAl_2_O_4_ (JCPDS-No. 34-0379) standard patterns were shown at the bottom.

The compositions of the typical Sr_4_Al_14_O_25_:0.014Mn^4+^, *x*B^3+^, 0Na^+^, (0 ≤ *x* ≤ 1.6) samples were displayed in Fig. 3a. When no flux is added, the compound Sr_4_Al_14_O_25_ is not formed, and instead, the two phases SrAl_2_O_4_ (JCPDS-No. 34-0379) and SrAl_12_O_19_ (JCPDS-No. 80-1195) appear. When boric acid is added, for instance at *x* = 0.2, the sample turns into a single phase of Sr_4_Al_14_O_25_. With increase of *x* from 0.2 to 1.2, XRD patterns of phosphors agree well with that of standard Sr_4_Al_14_O_25_ (JCPDS-No. 52-1876). Further increment of boric acid content will induce an extra impurity phase of SrAl_2_O_4_. Because boric acid has lower melting temperature, it (after being homogeneously dispersed throughout the sample) will be the first component in the mixture to melt at high temperature. This can promote the immigration of Sr and Al ions, for instance by diffusion, and increase the possibility that the ions encounter, and thus accelerate the crystallization process. Excess boron will dilute the content of Sr and Al ions in the sample and it is therefore not beneficial for the formation of the phase Sr_4_Al_14_O_25_. According to the emission spectrum (Fig. 7b), it is concluded that the optimum *x* of B^3+^ is 0.8. Comparison of B^3+^ dopant content in Sr_4_Al_14_O_25_:Mn^4+^, *x*B^3+^ is shown in Table 1. The compositions of the typical Sr_4_Al_14_O_25_:0.014Mn^4+^, 0.8B^3+^, *y*Na^+^, (0 ≤ *y* ≤ 3) samples were displayed in Fig. 3b. With increase of *y* from 0 to 2, XRD patterns of phosphors agree well with that of standard Sr_4_Al_14_O_25_ (JCPDS-No. 52-1876). Further increment of Na^+^ content will induce an extra impurity phase of SrAl_2_O_4_.

**Table 1:** Comparison of B^3+^ dopant content in Sr_4_Al_14_O_25_:Mn^4+^, *x*B^3+^

Range of *x*	Optimum value of *x*	Ref.
—	0.4	12
—	0.54	14
0–3.5	0.7	15
0–1.6	0.8	This work

**Fig. 4:**
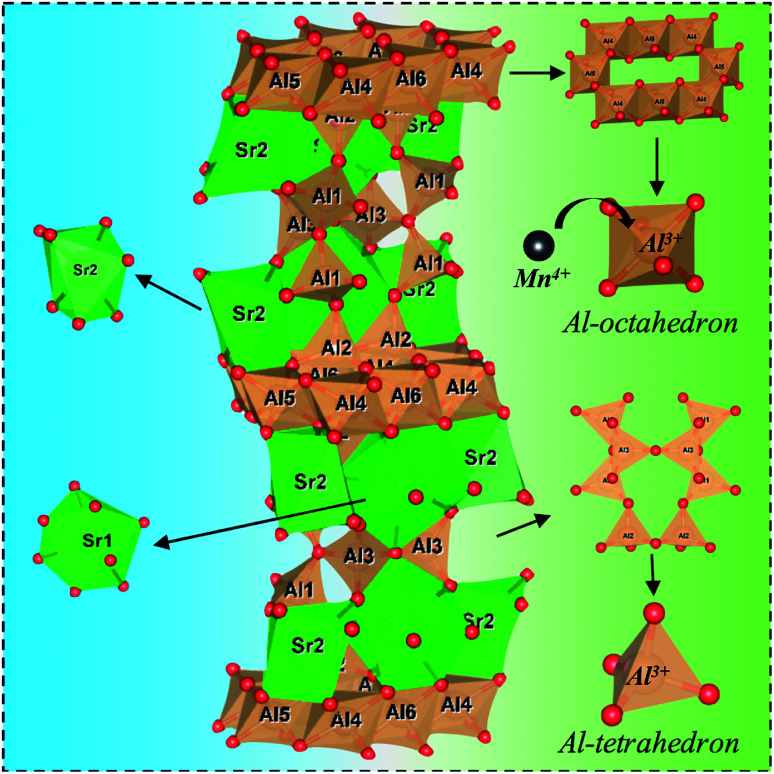
Schematic illustration of crystal structure of Sr_4_Al_14_O_25_. Black ball: Mn; Green ball: Sr; red ball: O; and brown ball: Al. Brown polyhedron: AlO_6_-octahedron/AlO_4_-tetrahedron; green polyhedron: SrO_7_/SrO_8_.

Fig. 4 shows a schematic of the Sr_4_Al_14_O_25_ crystal structure. The space group of Sr_4_Al_14_O_25_ is *Pmma* and orthorhombic. The networks of orthorhombic Sr_4_Al_14_O_25_ are built by one layer of the octahedral anion groups (AlO_6_) and several layers of tetrahedral anion groups (AlO_4_) alternatively. There are three types of AlO_6_ octahedrons (Al(4)O_6_, Al(5)O_6_ and Al(6)O_6_) and three types of AlO_4_ tetrahedron (Al(1)O_6_, Al(2)O_6_ and Al(3)O_6_). Compared with the strong covalence effect of the AlO_4_ tetrahedron, a little weak polarization field of the AlO_6_ octahedron is more suitable for Mn^4+^ incorporating. In addition, the Mn^4+^ ion always experiences a strong CF due to its high effective positive charge with the result that the emission spectrum is always dominated by the sharp emission line corresponding with the spin-forbidden ^2^E_g_ → ^4^A_2g_ transition.^16^

### Luminescence property

**Fig. 5:**
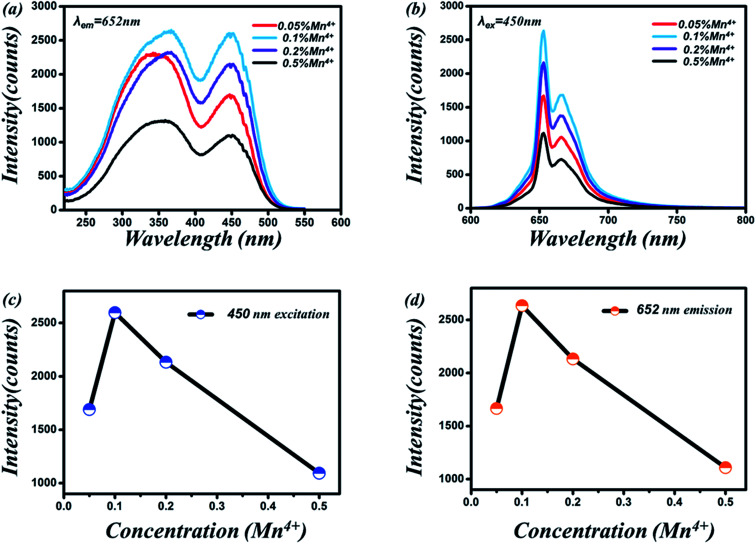
(a) PLE spectrum of Sr_4_Al_14_O_25_:0.8B^3+^, 2Na^+^, *z*Mn^4+^ (0.05% ≤ *z* ≤ 0.5%) phosphor monitored at 652 nm. (b) PL spectrum of Sr_4_Al_14_O_25_:0.8B^3+^, 2Na^+^, *z*Mn^4+^ phosphor excited by 450 nm blue light. (c) Dependence of Mn^4+^ PLE intensity on dopant concentration for Sr_4_Al_14_O_25_:0.8B^3+^, 2Na^+^, *z*Mn^4+^ phosphor. (d) Dependence of Mn^4+^ PL intensity on dopant concentration for Sr_4_Al_14_O_25_:0.8B^3+^, 2Na^+^, *z*Mn^4+^ phosphor.

Fig. 5a show the PLE spectrum of Sr_4_Al_14_O_25_:0.8B^3+^, 2Na^+^, *z*Mn^4+^ (0.05% ≤ *z* ≤ 0.5%) phosphor monitored at 652 nm. Fig. 5b show the PL spectrum of Sr_4_Al_14_O_25_:0.8B^3+^, 2Na^+^, *z*Mn^4+^ (0.05% ≤ *z* ≤ 0.5%) phosphor excited at 450 nm. Fig. 5c and d visually show the excitation and emission intensity of Mn^4+^ on dopant concentration for Sr_4_Al_14_O_25_:0.8B^3+^, 2Na^+^, *z*Mn^4+^ phosphor, respectively. As we can see, the optimum molar concentration of Mn^4+^ in Sr_4_Al_14_O_25_:0.8B^3+^, 2Na^+^, *z*Mn^4+^ in this work was 0.1 mol%. The photoluminescence emission spectrum of the phosphor presented a double-peak structure between 600 and 700 nm with two strong bands at about 654 and 664 nm, which were attributed to the ^2^E → ^4^A_2_ transition of Mn^4+^ ions and a phonon sideband transition, respectively.^13^

**Fig. 6:**
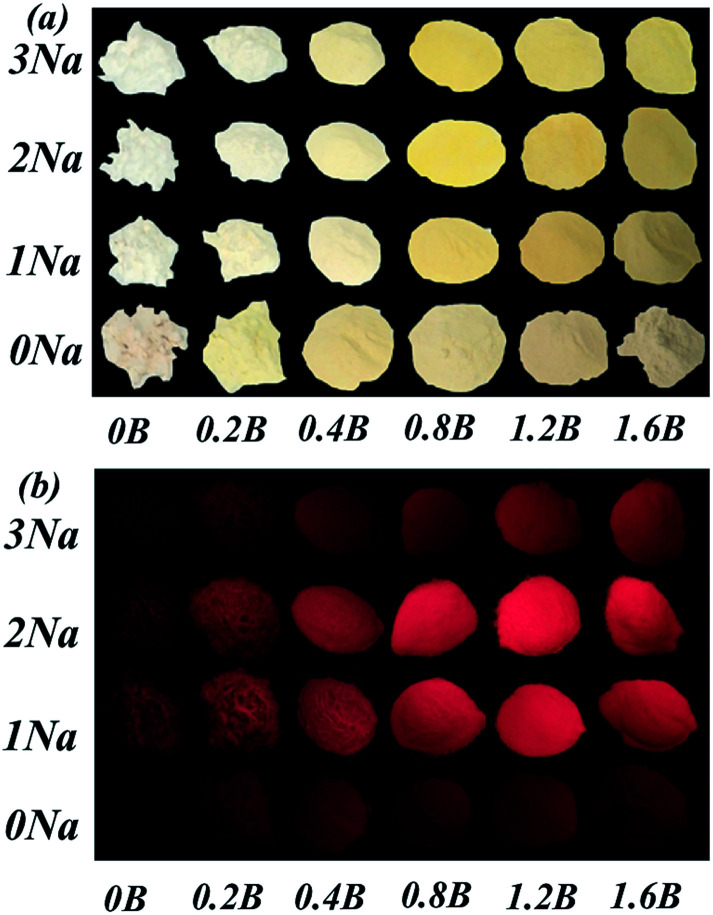
(a) Photographs of Sr_4_Al_14_O_25_:0.014Mn^4+^, *x*B^3+^, *y*Na^+^ (0 ≤ *x* ≤ 1.6, 0 ≤ *y* ≤ 3) under natural sunlight. (b) Photographs of Sr_4_Al_14_O_25_:0.014Mn^4+^, *x*B^3+^, *y*Na^+^ under 365 nm UV light.

**Fig. 7:**
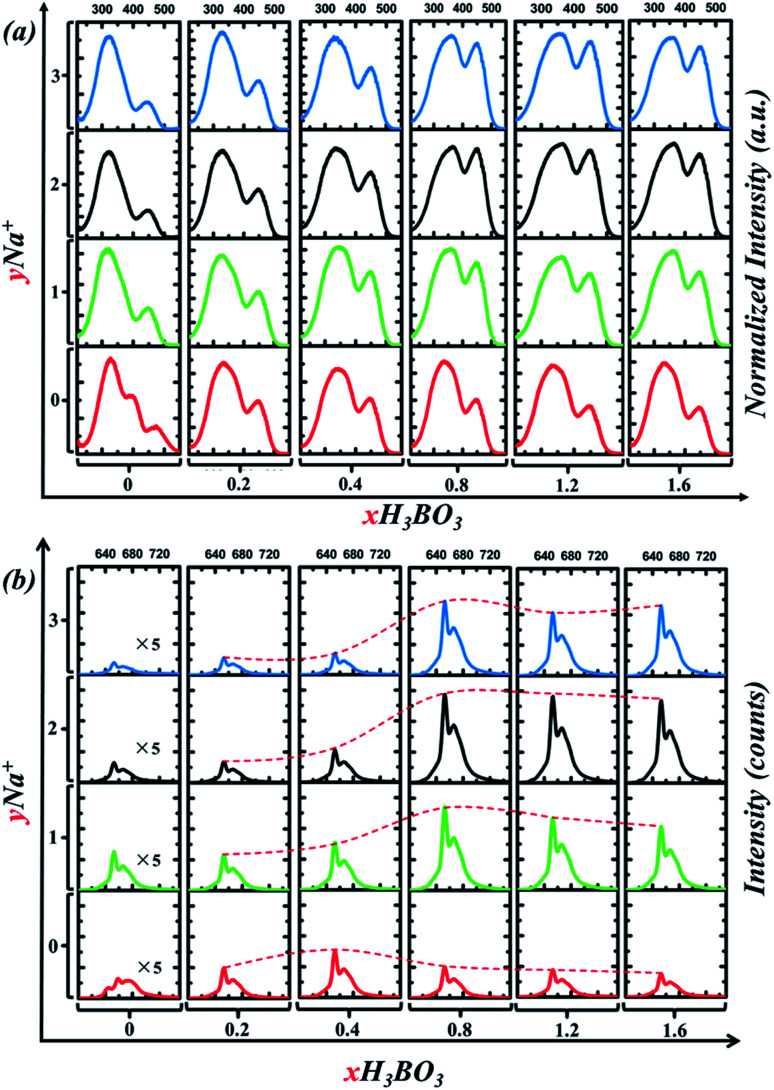
(a) PLE spectrum of Sr_4_Al_14_O_25_:0.014Mn^4+^, *x*B^3+^, *y*Na^+^ phosphor monitored at 652 nm. (b) PL spectrum of Sr_4_Al_14_O_25_:0.014Mn^4+^, *x*B^3+^, *y*Na^+^ phosphor excited by 450 nm blue light.

After obtained optimum Mn^4+^ doping concentration, we adjusted the amount of B^3+^ and Na^+^. Fig. 6a and b show the photographs of Sr_4_Al_14_O_25_:0.014Mn^4+^, *x*B^3+^, *y*Na^+^ (0 ≤ *x* ≤ 1.6, 0 ≤ *y* ≤ 3) under natural sunlight and 365 nm UV light, respectively. Along with Na^+^ ions and B^3+^ doping concentrations increasing, the color of Sr_4_Al_14_O_25_:0.014Mn^4+^, *x*B^3+^, *y*Na^+^ phosphors under natural sunlight change from light yellow to bright yellow. This might be ascribed to the Na^+^ and B^3+^ ions enhanced absorption band (220–500 nm), especially in the region of 400–500 nm, as illustrated in Fig. 7a.

In detail, Fig. 7a shows PLE spectrum of Sr_4_Al_14_O_25_:0.014Mn^4+^, *x*B^3+^, *y*Na^+^ phosphor monitored at 652 nm. In order to compare the relative changes of the excitation bands, the PLE intensity is normalized. When no Na^+^ is added, with *x* of B^3+^ increases from 0.2 to 1.6, the relative magnitude of excitation bands varies little. Furthermore, the optimum *x* of B^3+^ is 0.4, not 0.8, according to Fig. 7b. When Na^+^ is added, for instance at *y* = 1, the intensity of the blue excitation band (400–500 nm) has been dramatically increased, as shown in Fig. 7a. In Fig. 7b, we can see that the optimum *x* of B^3+^ turns to 0.8 from 0.4. When *x* of B^3+^ is fixed at 0.8, the emission intensity increased first, reached the maximum (*y* = 2), and then decreased with the increase of Na^+^ content. Cross experiment can clearly found that the optimal combination is *x* = 0.8, *y* = 2. To illustrate the difference between our experimental results and Meng's,^12^ the detail differences are listed in Table 2. As we can see the amount of doping Na^+^ and B^3+^ is observably different. As Fig. 7a shown, when *x* = 0.4, the addition of Na^+^ does not significantly affect the relative intensity between the two excitation bands. This is why Meng didn't report the asynchronous increase of excitation bands. This asynchronous increase of excitation bands, however, allows the phosphor to be more easily excited by blue light. As Fig. 7b shows, the PL emission intensity excited by 450 nm blue light sharply increased 3 times when *x* of B^3+^ turns to 0.8 from 0.4 (when *y* = 2). Therefore, addition of B^3+^ and Na^+^ ions in Sr_4_Al_14_O_25_:0.014Mn^4+^, *x*B^3+^, *y*Na^+^ can significantly improve its visible light excitation efficiency in the spectral range of 400–500 nm so that it can be incorporated as a red component into blue chip-based LED applications for plant growth.

**Table 2:** Comparison of dopant content in Sr_4_Al_14_O_25_:*x*B^3+^, *y*Na^+^, *z*Mn^4+^

Range of *x*	Optimum value of *x*	Range of *y*	Optimum value of *y*	Optimum value of *z*	Excitation bands	Ref.
0.4	—	0.05	0–0.09	0.01	Synchronous increase	11
0.8	0–1.6	2	0–3	0.014	Asynchronous increase	This work

**Fig. 8:**
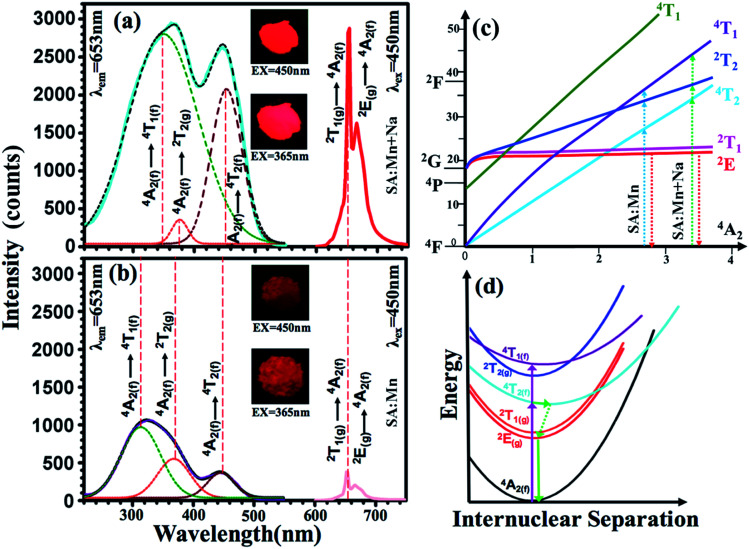
(a) PL (EX = 450 nm) and PLE (EM = 652 nm) spectra of Sr_4_Al_14_O_25_:0.014Mn^4+^, 2Na^+^, 0.8B^3+^ and (b) Sr_4_Al_14_O_25_:0.014Mn^4+^, 0.8B^3+^ respectively; (c) Tanabe–Sugano energy diagram of a 3d^3^ system in an octahedral crystal field; (d) configurational coordinate diagram for Mn^4+^ ions in Sr_4_Al_14_O_25_ hosts.

Fig. 8a and b show the photoluminescent excitation and emission spectra of the Sr_4_Al_14_O_25_:0.014Mn^4+^, 0.8B^3+^ based phosphors with or without co-incorporating Na^+^. The fluorescent intensities of the phosphors excited at 450 nm reached a maximum at *x* = 0.8, *y* = 2 and *z* = 0.014, and the strongest emission intensity of Sr_4_Al_14_O_25_:0.8B^3+^, 2Na^+^, 0.014Mn^4+^ sample were increased by 700% compared with Sr_4_Al_14_O_25_:0.8B^3+^, 0.014Mn^4+^ without Na^+^ co-doping. The photographs of the Sr_4_Al_14_O_25_:0.014Mn^4+^, 0.8B^3+^, 2Na^+^ sample and Sr_4_Al_14_O_25_:0.014Mn^4+^, 0.8B^3+^ sample exposed to 450 nm blue light and 365 nm UV light are shown in the insert of the Fig. 8a and b. After the incorporating of sodium ions, the brightness of phosphor becomes larger. We can find that the excitation spectra are consists of three conjoint bands by multi-peaks fitting, which located from near UV region to visible blue region. These three bands are corresponding to ^4^A_2_ → ^4^T_2_, ^4^A_2_ → ^2^T_2_ and ^4^A_2_ → ^4^T_1_ transition, respectively. Though according to the spin selection rule of Δ*S* = 0, the transitions between ^4^T_2_, ^4^T_1_ and ground ^4^A_2_ levels are spin-allowed, the spin-forbidden transition ^4^A_2_ → ^2^T_2_ is still be found in our result and other Mn-incorporated phosphors, such as CaAl_12_O_19_:Mn^4+^,^17^ SrMgAl_10_O_17_:Mn^4+^,^18^ Mg_2_TiO_4_:Mn^4+^,^10^ Li_2_Mg_3_SnO_6_:Mn^4+^,^19^ Ba_2_TiGe_2_O_8_:Mn^4+^,^20^ and Na_2_MgAl_10_O_17_:Mn^4+^.^21^ By fitting the peaks of the excitation spectra, we found that all the excitation peaks have a red shift as the sodium incorporated.

### Crystal filed strength calculation

The values of *D*_q_, *B* and *C* can be calculated based on experimentally determined energy levels using the following equations:^5^

1 *D*_q_ = *E*(^4^T_2g_ − ^4^A_2g_)/10

2
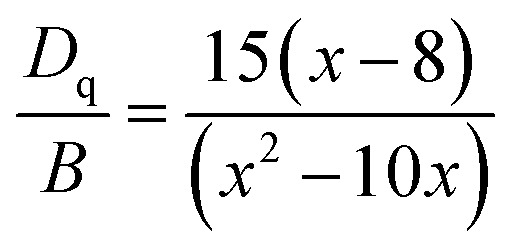
where *D*_q_ represents the crystal field strength and the parameter *x* is defined as3
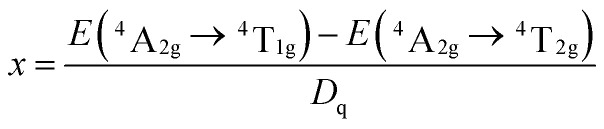

4 *E*(^2^E_g_ − ^4^A_2g_)/*B* = 3*C*/*B* + 9 − 9*B*/*D*_q_

From Fig. 8a, the energy levels of ^4^T_2_, ^4^T_1_ and ^2^E_g_ in the Sr_4_Al_14_O_25_:0.014Mn^4+^, 0.8B^3+^, 2Na^+^ host were determined at 22 124, 28 986 and 15 314 cm^−1^, respectively. From Fig. 8b, the energy levels of ^4^T_2_, ^4^T_1_ and ^2^E_g_ in the Sr_4_Al_14_O_25_:0.014Mn^4+^, 0.8B^3+^ host were determined at 22 321, 30 769 and 26 667 cm^−1^, respectively. From eqn (1)–(4), the crystal field parameters of *D*_q_, *B*, *C* in the Sr_4_Al_14_O_25_:0.014Mn^4+^, 0.8B^3+^, 2Na^+^ were calculated to be 2212, 644 and 3735 cm^−1^, respectively. The crystal field parameters of *D*_q_, *B*, *C* in the Sr_4_Al_14_O_25_:0.014Mn^4+^, 0.8B^3+^ were calculated to be 2232, 830 and 3540 cm^−1^, respectively. Once these parameters have been determined, the energies of all other states such as ^2^T_1_, ^2^A_1_ and ^4^T_1_ can be theoretically predicted by:

5 *E*(^2^T_1_ − ^2^E_g_) = 66*B*^2^/(10*D*_q_)

6 *E*(^2^A_1_ − ^4^A_2_) = 10*D*_q_ + 4*B* + 3*C*

7 *E*(^4^T_1_(P) − ^4^A_2_) = 15*D*_q_ + 7.5*B* − 0.5[100*D*_q_^2^ − 180*D*_q_*B* + 225*B*^2^]^1/2^

The crystal field parameters and the energies of states in Sr_4_Al_14_O_25_:0.014Mn^4+^, 0.8B^3+^, 2Na^+^ and Sr_4_Al_14_O_25_:0.014Mn^4+^, 0.8B^3+^ crystal lattices are summarized in the Table 3. As shown in Fig. 8c, the dependence of energy levels of Mn^4+^ on crystal field strength can be illustrated by Tanabe–Sugano energy diagram. The ^2^E_g_ levels are almost parallel to the ground state ^4^A_2_, which results that the location of the emission peak is difficult to be influenced by crystal field strength. While, the energy gap between ^4^T_1_ (or ^4^T_2_) levels and ground state ^4^A_2_ can be changed by variation of the crystal field strength. The electron transition schematic diagrams are shown in the Fig. 8c with blue and green dot lines. The value of *D*_q_/*B* increased to 3.43 from 2.69 as the Na^+^ ions addition. There is a difference in the asynchronous increases of the near UV and visible absorption bands. The increase of the excitation intensity at visible blue region is much larger than at near UV region, even both excitation intensity at these two regions are almost equal. Na^+^ compounds are well known fluxes in solid state synthesis. However, the shapes of the excitation bands cannot be changed by fluxes and meanwhile the redshift of the excitation is a change on the luminous mechanism instead of fluxes effect.

**Table 3:** Crystal field parameters and energies of states in Sr_4_Al_14_O_25_:0.014Mn^4+^, 0.8B^3+^, 2Na^+^ and Sr_4_Al_14_O_25_:0.014Mn^4+^, 0.8B^3+^ crystal lattices

	Sr_4_Al_14_O_25_:0.014Mn^4+^, 0.8B^3+^, 2Na^+^ (cm^−1^)	Sr_4_Al_14_O_25_:0.014Mn^4+^, 0.8B^3+^ (cm^−1^)
*D*_q_	2212	2232
*B*	644	830
*C*	3735	3540
*D*_q_/*B*	3.43	2.69
^4^A_2_ → ^4^T_2_	22 124	22 321
^4^A_2_ → ^2^T_2_	26 455	26 667
^4^A_2_ → ^4^T_1_	28 985	30 769
^2^E_g_ → ^4^A_2_	15 314	15 314
^2^E_g_ → ^2^T_1_	1238	2039
^4^A_2_ → ^2^A_1_	35 905	36 264
^4^A_2_ → ^4^T_1_	28 986	30 769

Fig. 8d shows a schematic diagram of a process of photoluminescence. The ^2^E_g_, ^2^T_1_, ^2^T_2_ and ^4^A_2_ levels are derived from the t^3^_2_ electronic orbital, whereas the ^4^T_1_ and ^4^T_2_ levels are formed from another t^2^_2_e orbital, resulting a displacement between the parabolas of ground state ^4^A_2_ and ^4^T_1_ (or ^4^T_2_). The electronics are excited from to ground state ^4^A_2_ to ^4^T_1_, ^4^T_2_ or ^2^T_2_ levels by radiation. Then, the excited electronics usually relax non-radiatively to ^2^E_g_ followed by the spin-forbidden ^2^E_g_ → ^4^A_2_ transition characterized by wide emission bands.

## Conclusions

A series of Sr_4_Al_14_O_25_:*x*B^3+^, *y*Na^+^, *z*Mn^4+^ red phosphors were synthesized by a high-temperature solid-state reaction method at 1400 °C for 6 hours in the air. The fluorescent intensities of the phosphors excited at 450 nm reached a maximum at *x* = 0.8, *y* = 2 and *z* = 0.014, and the strongest emission intensity of Sr_4_Al_14_O_25_:0.8B^3+^, 2Na^+^, 0.014Mn^4+^ sample were increased by 700% compared with Sr_4_Al_14_O_25_:0.8B^3+^, 0.014Mn^4+^ without Na^+^ co-doping. In comparison with Mn^4+^ single incorporated phosphor, Sr_4_Al_14_O_25_:0.014Mn^4+^, 0.8B^3+^, 2Na^+^ shows greater advantage of promising application incorporated as a red component into blue chip-based LED for plant growth because of much stronger absorption at blue light region and enhanced red emission. The prepared phosphors could be efficiently excited by both near-UV light and the commercially available blue light of LED chips at 450 nm.

## Conflicts of interest

There are no conflicts to declare.

## Supplementary Material
